# A 4-year follow-up of the need for orthodontic treatment using the Dental Aesthetic Index-DAI: an exploratory analysis

**DOI:** 10.1590/1807-3107bor-2025.vol39.007

**Published:** 2025-01-20

**Authors:** Silvia Amelia Scudeler VEDOVELLO, Ana Letícia Mello de CARVALHO, Diego Patrik Alves CARNEIRO, Marcelo de Castro MENEGHIM

**Affiliations:** (a) Universidade Estadual de Campinas – Unicamp, Piracicaba Dental School, Department of Health Sciences and Child Dentistry, Piracicaba, SP, Brazil.; (b) Universidade Estadual de Campinas – Unicamp, Piracicaba Dental School, Department of Community Dentistry, Piracicaba, SP, Brazil.

**Keywords:** Malocclusion, Dentition, Mixed, Dentition, Permanent

## Abstract

This study aim was to evaluate the need for orthodontic treatment of mixed to permanent dentition using the Dental Aesthetic Index (DAI) in a 4-year follow-up. A longitudinal study was conducted with 353 children in the stages from mixed (T1) to permanent (T2) dentition. The need for orthodontic treatment was assessed using the DAI categorized into: DAI 1 (absence of malocclusion and orthodontic treatment need; DAI ≤ 25); DAI 2 (malocclusion is defined and elective orthodontic treatment is needed; DAI = 26 to 30); DAI 3 (severe malocclusion and a desirable orthodontic treatment need; DAI = 31 to 35) and DAI 4 (severe malocclusion and a mandatory orthodontic treatment need; DAI ≥ 36). The Bowker symmetry test was used to determine agreement in the categorization of DAI at T1 and T2, with a significance level of 5%. The results showed a significant disagreement in the need for orthodontic treatment between T1 and T2 (p<0.05). In 34.6% of children evaluated in T1, the same need for orthodontic treatment was maintained in T2. According to the DAI, in 60.8% of the children, the need decreased, and in 39.2% their need for orthodontic treatment increased. This preliminary longitudinal study using DAI, showed a decrease in malocclusion and need for orthodontic treatment as the dentition transitioned from mixed to permanent occurred. This finding has valuable implications for epidemiological data in orthodontics.

## Introduction

Divergences in the prevalence and severity of malocclusion and, consequently, in need for orthodontic treatment can be attributed to variations in the indices selected for assessing population data.^1-5^Epidemiological indices such as the Dental Aesthetic Index (DAI)^6,7^ or the Index of Orthodontic Treatment Need (IOTN)^1^ standardize malocclusion assessment by weighing occlusal characteristics to generate a score. In particular, the DAI and IOTN measure treatment needs in individuals or groups to ensure that individuals with the greatest need receive treatment and to help with orthodontic workforce planning.^1-5,8^

The prevalence of malocclusion in primary and permanent dentition is well-documented. The World Health Organization (WHO) uses the ages of five and 12 to assess the prevalence of malocclusion, as these ages correspond to key stages in the development of dentition and occlusion. Although only a few epidemiological studies have examined mixed dentition,^9-12^ the majority have utilized the DAI.^13-19^ The prevalence of malocclusion in this stage varies widely, ranging from 32.20% to 82.50%.^14,20-25^ This variability can largely be attributed to a lack of standardization in assessment tools and data collection methods, which can result in underestimation or overestimating outcomes.^5,24^

Another important aspect to be highlighted is that, throughout the phase of mixed dentition, certain malocclusions have the potential to self-correct or reduce their severity, which may explain the variations noted among the diverse occlusal stages. Thus, identifying conditions that can self-correct during development is important, as it may prevent unnecessary orthodontic treatment. It is also necessary to acknowledge that the physiological changes during the mixed dentition phase can create challenges in establishing normative occlusion. However, many interceptive orthodontic treatments are performed during this period, underscoring the importance of accurate epidemiological data.^5,24^

Thus, longitudinal studies that follow the transition from mixed to permanent dentition are considered necessary in Orthodontics, as they allow a deeper understanding of changes in occlusion. Furthermore, the divergences between data in the literature and the need to understand the limitations of the DAI justify this study. Therefore, in this study a longitudinal exploratory analysis was performed to monitor the need for orthodontic treatment during the mixed to permanent dentition using the DAI.

## Methods

### Study design and sample

This study received approval from the Human Research Ethics Committee of Brazil (#52809521.2.0000.5385). Parents/caregivers signed a term of informed consent authorizing their children’s participation.

A longitudinal observational study was conducted with children in the stages from mixed to permanent dentition, in a 4-year follow-up. The study was conducted with children enrolled in public schools in Brazil, in a city with an estimated population of 135,506 inhabitants (IBGE-2020) and a high Human Development Index (HDI) (0.781).

The first assessment was conducted in 2018 and included 785 children in the mixed dentition stage (T1), between 8 and 10 years old. The mixed dentition was determined considering the dental age, proposed by Van der Linden^26^ and based exclusively on oral clinical examination. The exclusion criteria included children undergoing Orthodontic treatment at that time, primary dentition, complete permanent dentition, and craniofacial syndromes.

All children evaluated in 2018 were contacted in 2022. Those who reported current or previous orthodontic treatment, with parents or guardians who did not authorize the examination, and who did not respond to contact were excluded. Three hundred fifty-two (44.9%) children were assessed. The children evaluated had permanent dentition (up to appearance of the permanent second molars).

### Clinical evaluation

The malocclusion was clinically evaluated inside the schools under natural light, by a calibrated evaluator.

In accordance with Dental Aesthetic Index (DAI)^6,7,27^ recommendations, measurements were taken in millimeters with the teeth in centric occlusion and the probe parallel to the occlusal plane. DAI includes ten parameters of dentofacial anomalies related to the following clinical and esthetic aspects: the number of visibly absent teeth, anterior crowding, anterior spacing, midline diastema, maxillary anterior misalignment, maxillary anterior horizontal overlap, mandibular anterior horizontal overlap, anterior open and, anteroposterior molar relationship.^6,7,27^

After evaluating the occlusal traits, the DAI score was analyzed using the sum of the scores of each characteristic added to a constant value. This sum leads to a classification that identifies the orthodontic treatment need of individuals as determined by the severity of the occlusal pathologies present: Grade 1 indicates the absence of malocclusion and orthodontic treatment need (DAI ≤ 25); Grade 2 indicates malocclusion is defined and elective orthodontic treatment is needed (DAI = 26 to 30); Grade 3 indicates severe malocclusion and a desirable orthodontic treatment need (DAI = 31 and 35) and Grade 4 indicates severe malocclusion and a mandatory orthodontic treatment need (DAI ≥ 36).^6,7,27^

### Training and calibration

The training and calibration process was carried out, and the same method was adopted at both time intervals of assessment (T1 and T2). The training consisted of a theoretical and practical discussion to ensure a deep understanding of the occlusal conditions evaluated. In the calibration phase, inter- and intra-examiner agreements were estimated using the intraclass correlation coefficient for each occlusal condition, with an acceptable limit value greater than 0.92 in the first (T1) and 0.97 in the second assessment (T2).

### Statistical analysis

Initially, descriptive analyses of the data were performed. The concordance in the classification of the need for orthodontic treatment determined by the DAI and evaluated in the mixed and permanent dentition was performed using the Bowker symmetry test, with a significance level of 5%. R program (R Foundation for Statistical Computing, Vienna, Austria) performed the analysis.

## Results

Table shows the variation in the DAI categories from mixed dentition (T1) to permanent dentition (T2). The results showed a significant disagreement in the need for orthodontic treatment between T1 and T2 (p < 0.05). The results showed that of the 352 children evaluated in T1, 122 (34.6%) maintained the same need for orthodontic treatment in T2. Of the 230 (65.4%) children who changed their orthodontic status, 140 (60.8%) showed decreased need, and 90 (39.2%) increased their need for orthodontic treatment, according to the DAI.

**Table 1:** Variation in DAI index category from mixed dentition to permanent dentition.

Variable	DAI in mixed dentition	DAI in permanent dentition				Total	p-value
		Grade 1	Grade 2	Grade 3	Grade 4		
Sample	Grade 1	46 (13,1%)	23 (6,5%)	20 (5,7%)	8 (2,3%)	97 (27,6%)	0,0007
	Grade 2	39 (11,1%)	18 (5,1%)	19 (5,4%)	8 (2,3%)	84 (23,9%)	
	Grade 3	17 (4,8%)	17 (4,8%)	15 (4,3%)	12 (3,4%)	61 (17,3%)	
	Grade 4	31 (8,8%)	19 (5,4%)	17 (4,8%)	43 (12,2%)	110 (31,3%)	
	Total	133 (37,8%)	77 (21,9%)	71 (20,2%)	71 (20,2%)	352 (100,0%)	
Diagonal sum (agreement)		122 (34,7%)					

DAI = 1: absence of malocclusion and need for orthodontic treatment; DAI = 2: Elective orthodontic treatment; DAI = 3: desirable orthodontic treatment; DAI = 4: mandatory orthodontic treatment.

## Discussion

This study aim was to conduct a longitudinal exploratory analysis of the need for orthodontic treatment using the DAI during the period of transition from mixed to permanent dentition. Cons et al.^6^ developed the DAI in the 1980’s, for the purpose of performing epidemiological evaluation of malocclusion in the population with permanent dentition. The index assesses the need for orthodontic treatment based on the severity of malocclusion and combines clinical and esthetic parameters that result in scores divided into four grades.^28,29^

The study was not without challenges. Although the DAI is a consolidated instrument, it may have limitations when used to evaluate malocclusion in mixed dentition. Some conditions of occlusion, such as median diastema, for example, are common at this stage, however, they can lead to an overestimation of the prevalence of malocclusion in mixed dentition when using the DAI as a measure.^15,16^ Consequently, its accuracy in determining the prevalence of malocclusion in mixed dentition may be compromised, whereas, from an epidemiological perspective, there is no specific instrument to evaluate malocclusion in mixed dentition.^5^To the best of our knowledge, this study is the first to contribute to orthodontics by investigating the longitudinal application of DAI in overseeing occlusion and determining the necessity for orthodontic intervention.

Considering the study design, important aspects motivated the use of the index in the present longitudinal follow-up. In the literature, the DAI is widely used within the context of epidemiology. Moreover, it provides a score for the severity of malocclusion and the need for orthodontic treatment. Furthermore, essential aspects that must be intercepted in mixed dentition, such as posterior crossbite, are ignored, thus underestimating the need for treatment at this stage. One suggestion would be to adapt the DAI for assessing the phase of development.

Our results demonstrated a statistically significant change in the severity of malocclusion and the demand for orthodontic treatment at the 4-year follow-up. When assessing the cohort of children, 34.7% exhibited the same level of malocclusion severity and need for orthodontic intervention in permanent dentition. Conversely, the majority, 65.4% of the children showed alterations in their occlusal status during the follow-up period. Among these, 60.8% experienced decreased severity and necessity for orthodontic treatment. The design of the index could serve as an explanation for the decrease in severity and the necessity of orthodontic intervention.

Our study raises important questions. The data suggested that conditions such as midline diastema and spacing may have led to overestimating the need for intervention during mixed dentition. We also considered the potential implications of specific situations, such as mandibular arch crowding, anterior open bite, and self-correction, which may indicate a decrease in the severity of malocclusion. The esthetic conditions have a significant weight in the final DAI score, leading to overestimating the need for orthodontic treatment at this stage.

Therefore, the findings suggested that the DAI could be influenced by the characteristics of mixed dentition, which could result in an overestimation of malocclusion severity and the necessity for orthodontic intervention.^13,30^ Recognizing the limitations of this measure is essential for making well-informed choices regarding its application. Consequently, when dealing with mixed dentition, orthodontists are encouraged to exercise caution in applying the criteria or consider using a different set of criteria that offer more precise assessments. Furthermore, a longitudinal examination of the DAI has the potential to uncover key variables that impact the progression of malocclusion, thus aiding in the formulation of more efficient preventive and therapeutic approaches, and potentially validating its usefulness in epidemiological investigation of malocclusion.

This preliminary longitudinal study further highlights the necessity of creating an epidemiological tool for mixed dentition to ascertain malocclusion and orthodontic intervention accurately. The ability to monitor alterations in disease rates and occlusal configurations over time is essential and directly applicable to allocating resources during the optimal period for orthodontic management.

## Conclusion

This preliminary longitudinal study showed a decrease in malocclusion and need for orthodontic treatment as the dentition transitioned from mixed to permanent stage, using DAI, which has valuable implications for epidemiological data in orthodontics.
